# Longitudinally adaptive assessment and instruction increase numerical skills of preschool children

**DOI:** 10.1073/pnas.2002883117

**Published:** 2020-10-26

**Authors:** Stephen W. Raudenbush, Marc Hernandez, Susan Goldin-Meadow, Cristina Carrazza, Alana Foley, Debbie Leslie, Janet E. Sorkin, Susan C. Levine

**Affiliations:** ^a^Department of Sociology, University of Chicago, Chicago, IL 60637;; ^b^Harris School of Public Policy, University of Chicago, Chicago, IL 60637;; ^c^Committee on Education, University of Chicago, Chicago, IL 60637;; ^d^Department of Education and Child Development, National Opinion Research Center, University of Chicago, Chicago, IL 60637;; ^e^Department of Psychology, University of Chicago, Chicago, IL 60637;; ^f^UChicago STEM Education, University of Chicago, Chicago, IL 60637

**Keywords:** preschool instruction, adaptive assessment, social inequality, randomized control trials, mathematics education

## Abstract

Social inequality in mathematical skill is apparent at kindergarten entry and persists during elementary school. To level the playing field, we trained teachers to assess children’s numerical and spatial skills every 10 wk. Each assessment provided teachers with information about a child’s growth trajectory on each skill, information designed to help them evaluate their students' progress, reflect on past instruction, and strategize for the next phase of instruction. A key constraint is that teachers have limited time to assess individual students. To maximize the information provided by an assessment, we adapted the difficulty of each assessment based on each child’s age and accumulated evidence about the child’s skills. Children in classrooms of 24 trained teachers scored 0.29 SD higher on numerical skills at posttest than children in 25 randomly assigned control classrooms (*P* = 0.005). We observed no effect on spatial skills. The intervention also positively influenced children’s verbal comprehension skills (0.28 SD higher at posttest, *P* < 0.001), but did not affect their print-literacy skills. We consider the potential contribution of this approach, in combination with similar regimes of assessment and instruction in elementary schools, to the reduction of social inequality in numerical skill and discuss possible explanations for the absence of an effect on spatial skills.

## Significance

Socioeconomic disparities in math proficiency are observable when children enter kindergarten, and these disparities persist through the school years. Research suggests that overall proficiency at kindergarten entry depends upon specific skills that all normally developing children age 3 to 5 y can learn. We therefore designed a procedure that enables teachers to assess the skills of each child and tailor instruction to child-specific levels of skill. The procedure is iterative: Assess, teach, reassess, and teach, with three assessments per school year. We found that children in classrooms randomly assigned to this procedure gained substantially more in their numerical proficiency than did children in control classrooms. The program did not delay growth in print literacy and increased verbal proficiency.

As early as kindergarten, children from low-income families trail behind their middle- to higher-income peers in mathematical knowledge (1–7). These disparities tend to persist (3), and math knowledge prior to the start of kindergarten predicts children’s future academic success not only in mathematics (8, 9), but also in reading (8). Social inequality in math skill reinforces social inequality in economic opportunity (10, 11).

We reasoned that an important step in narrowing this achievement gap is to enhance math instruction in preschool classrooms serving low-income families (12–15). Because even very young children vary substantially in their math skills, we anticipated that, to be successful, preschool teachers must tailor their instruction to the varied skills of their students (2, 16). This tailoring requires that teachers gain detailed knowledge about the skills each child needs to develop and about the child’s progress on those skills. We therefore designed a system of longitudinally adaptive assessment and instruction that enables teachers to assess each child’s numerical and spatial skills and to enact child-specific instructional strategies to improve those skills. This process iterates three times during the school year. We regard such iteratively adaptive assessment and instruction as a dynamic instructional regime (17) analogous to a dynamic treatment regime in medicine (18).

Based on accumulating evidence that effects of early math interventions fade over time (19, 20), we do not anticipate that our preschool intervention would be sufficient to reduce the achievement gap observable when children enter secondary school. Rather, we reason that, if effective, this instructional regime would raise the whole distribution of math proficiency among low-income children at kindergarten entry, creating potential for reducing inequality over the course of elementary school. However, to fully capitalize on this potential, we reason that their elementary school teachers, who themselves encounter children of widely varied skill, would enact a similar dynamic instructional regime. Recent research on elementary school interventions (21–23) supports this reasoning, as it suggests that such instructional regimes can produce substantial gains in learning for children whose skills vary widely.

More generally, we theorize that, at every stage of development, children are remarkably heterogeneous with respect to their knowledge and skill in key domains such as mathematics. Under the current education system, we ask teachers to promote the mathematical knowledge and skills of every child, but without adequate information about what children already know, what they need to know to advance to the next level, how to tailor instruction to push learning to the next level, or how well current efforts are working toward that end. We reason that, to substantially increase math learning and to eliminate disparities based on social origins, this kind of dynamic instructional regime would ideally continue seamlessly throughout the school years. In the current study, we take an essential step in establishing the potential of such a regime by testing its efficacy in the preschool years.

We first identified 12 mathematics skills that we regarded as key components of early numerical and spatial reasoning based on our review of the literature (4, 9, 24–32) and designed assessments of these skills that teachers could easily carry out in a classroom setting. To evaluate the validity of these assessments, we conducted a field test in which we administered over 300 items to 400 children. To set targets for children’s growth, we estimated the average kindergarten-entry math achievement of nonpoor students in a nationally representative sample—the Early Childhood Longitudinal Study–Kindergarten Cohort (ECLS-K) of 1998. We then equated this level of achievement with the measures in our study to set target levels of proficiency for each of our 12 skills. To make this system useful in practice, we constructed eight assessment booklets, each of which could be administered in about 15 min. The booklets varied in difficulty, enabling us to match the difficulty level of the booklet to the current estimate of a child’s skill level, thus efficiently using constrained time for the assessments. To recommend effective instructional approaches and design specific strategies tailored to varying skill levels, we reviewed literature on early math learning, collaborated with expert teachers and coaches, and conducted pilot tests. We then conducted a randomized trial, reported here, the following year.

To clarify the theoretical basis of this intervention, we briefly review the sources of early inequality in math achievement. Next, we define what we have called foundational skills and review what is known about how to use periodic assessments to tailor instruction to child-specific skill levels. Finally, we describe in detail what is meant by a dynamic instructional regime in early math and how this strategy connects with a life-course developmental framework for eliminating social inequality in mathematics.

## Understanding the Math Achievement Gap

Children’s early engagement in mathematical thinking in their home and school environments is linked to their mathematical development (2, 4, 33–35). However, there is wide variability in the amount and quality of the math talk that young children are exposed to at home (4), as well as at school (2). Furthermore, there is evidence from studies of the home environment that the amount and quality of math input children receive are positively related to family socioeconomic status (SES) (6, 16, 36–38). For example, 14- to 30-mo-olds from higher-SES families hear more number words spoken by parents in the home environment than children from lower-SES families (4). Further, 4-y-olds from higher-SES families receive numerical input involving larger numbers and a greater variety of mathematical activities, such as mathematically relevant games and toys, than do children from lower-SES families (6, 38).

If young children from low-SES families have comparatively limited opportunities to learn math at home, it is not surprising that they are at a disadvantage on a range of mathematical skills when they enter kindergarten (5, 39–41). Early socioeconomic disparities in children’s mathematical understanding are observable on tasks that are relatively complex and require high levels of language and conceptual knowledge (6, 42, 43). For example, we see greater disparities in understanding the cardinal meanings of number words than on rote counting and greater disparities on verbally demanding skills, such as solving word problems and number fact problems, than on calculations that are administered and responded to nonverbally (40, 44).

SES disparities in home math learning opportunities underscore the need for high-quality preschool math instruction for low-SES children. Yet, preschool instruction has not historically focused on math, as indexed by the low percentage of the total day that on average is devoted to math instruction (45, 46). Moreover, there is wide variability across preschool classrooms in math instructional content (2, 47–49). We sought to help preschool teachers understand the importance of early math, gain knowledge of the specific skills their children need to master, understand early math learning trajectories, and enact instructional strategies tailored to the varied levels of children’s skills.

## Foundational Mathematical Skills

Below we review evidence pointing to foundational math competencies for children 3 to 5 y of age. A set of guidelines for preschool math instruction in numerical and spatial domains published by the National Research Council (50) is consistent with this evidence. The 12 skills included in our assessment system were chosen to tap these foundational competencies and are shown in Table 1.

**Table 1.** Descriptions, number of items, and reliabilities (Cronbach’s alphas) for GoT Assessment tasks

Domain	Skill (α)	Tasks (No. items)
Numerical thinking	Cardinality (0.84)	Show how many objects are on a page using fingers; produce requested number of objects (10).
	Counting (0.88)	Point to and label objects with number words; count starting from 1; recite number sequence forward or backward from given number >1 (20).
	Written numeral knowledge (0.92)	Identify written numeral by name (11).
	Comparing and ordering sets (0.87)	Select which is more of two hands showing amounts 1 to 5; place cards with different numbers of fingers or dots in numerical order (12).
	Operations (0.86)	Add and subtract objects in a cup; add and subtract using stories about objects in a cup (15).
Spatial thinking	Shape knowledge (0.87)	Identify a given shape from a selection of 4; decide whether a shape is a triangle or not (33).
	Shape features (0.78)	Find shape that contains displayed part; find shape that has certain verbal criteria (15).
	Spatial Relationships (0.82)	Find picture displaying the same spatial relationship/orientation as model picture (20).
	Patterns and structure (0.85)	Copy pattern shown using identical shapes; predict next color shown in pattern (8); create same type of pattern shown using different shapes (8).
	Mental rotation (0.84)	Among three choices, find animal that faces a specified direction when rotated (20).
	Shape composition (0.90)	Combine shapes to fill outlines of bigger shapes (14).
	Mathematical vocabulary (0.91)	Find picture that displays verbally presented term (48).

### Foundational Numerical Skills.

Core numerical skills require fluent and flexible knowledge of the verbal count list (51, 52). Children learn to link the count list to the quantities that numerals represent by counting objects in a set and by hearing cardinal number words linked to the count list (e.g., “There are three frogs—one, two, three.”) (52–56). This cardinal number knowledge enables children to compare the exact magnitude of small sets that are too close in numerosity to differentiate using approximate number skills (57), order the sets, and carry out calculations involving multiple sets (58–62). Identification of written numerals enables children to perform these tasks with written, as well as verbal, number symbols (58, 63). These skills set the stage for more advanced numerical skills taught in elementary school (9, 24, 51), and disparities in these skills help explain socioeconomic disparities in the math achievement of children in elementary school and beyond (9). Teaching these skills effectively requires some knowledge of how early conceptual knowledge and procedural skills support learning numerical concepts and skills that develop later (64) and understanding how to advance the skills of young children who are at different points on established math learning trajectories (65).

### Foundational Spatial Skills.

Although the development of spatial skills in preschool is not as well understood as the early development of numerical skills, research highlights the importance of early spatial learning to later mathematical achievement (66–68). Skills recommended for preschool instruction involve knowing shapes and their features (50), the ability to mentally transform objects in space (25, 66), and the ability to construct larger shapes and structures from smaller shapes and structures (30). These skills involve developing an understanding of spatial relations between and within objects, as well as an ability to understand the orientations of objects (34, 35, 69).

A growing body of literature shows that early spatial thinking is related to early math skills (25, 28–30, 70, 71). In older children and adults, spatial skills predict Science, Technology, Engineering, and Mathematics achievement and career paths, even when controlling for numerical and verbal abilities (25, 72–76). Although there are socioeconomic disparities in foundational spatial skills (35, 39), spatial thinking can be improved with instruction and practice (67, 77, 78). Moreover, training children’s spatial thinking improves their numerical math skills (66, 67).

### Foundational Skills Spanning Numerical and Spatial Domains.

Some preschool math skills span both numerical and spatial domains. These include understanding patterns—which can be spatial, quantitative, or both—as well as understanding math vocabulary, which includes knowledge of words that refer to measurement, quantitative relations, and spatial relations. Patterning skill in preschool predicts later math achievement, even when controlling for other math knowledge (79, 80). Moreover, pattern-related interventions have been found to increase not only children’s patterning skills (81), but also their scores on broader measures of math achievement (82, 83). Children’s math and spatial vocabulary has also been shown to be strongly linked to their math and spatial skills (35, 84–86). For instance, preschool-age children’s understanding of mathematical language, including terms such as “more” and “less,” is positively related to their numerical skills, such as comparing and ordering numerals, above and beyond their basic number sense (85). Similarly, children’s use of spatial language predicts their performance on nonverbal spatial tasks, such as carrying out mental transformations, building block structures, and understanding spatial analogies (35).

## Addressing Heterogeneity in Students’ Math Proficiency

The fact that preschoolers vary substantially in their math proficiency presents teachers with the pedagogical challenge of meeting the heterogeneous learning needs of their students. One approach to addressing this challenge is to provide differentiated instruction tailored to students’ current proficiency on foundational skills. Critically, teachers need to know what students’ current proficiencies actually are to do this tailoring.

Research in elementary schools shows that teachers can acquire this information by periodically assessing students’ knowledge of foundational math skills and using the results to plan instruction tailored to the specific needs of each student. The Indiana Department of Education adopted a school-level intervention in which kindergarten to eighth grade teachers administered assessments four times during the school year and received detailed reports from the assessment vendors, who also provided guidance on how to tailor instruction to individual children. Konstantopoulos et al. (23) randomly assigned 35 schools to receive this intervention, with 24 schools randomly assigned to a control condition. Results showed a significant positive impact of the intervention on student math outcomes in grades 3 to 8 with an effect of about 20% of a SD, although no impact was detected on student math outcomes in grades K to 2. In another study, Connor et al. (21) randomly assigned 32 second-grade classrooms to a math intervention in which teachers used data from ongoing student assessments to plan individualized math instruction. Teachers administered assessments four times over the school year, each time regrouping students for instruction tailored to current student skill. This math intervention increased achievement by approximately 0.5 SD. Hassrick et al. (87) used a randomized lottery to assess the impact of a school-wide instructional regime based on frequent assessment and instructional planning in reading and math each year from kindergarten to grade 5. Large learning gains in grades K to 5 were sustained in middle school. These exciting findings encourage us to envision the potential positive effects of a coherent and sustained regime of iterative assessment and individualized planning across a broad range of grades. Reducing social inequality in math at entry to kindergarten would, in theory, be an essential step toward this goal in view of findings that math knowledge at kindergarten entry is predictive of long-term academic outcomes (3, 8). Our study addresses this goal by mobilizing findings from basic science about foundational early math skills to devise a dynamic regime of assessment and instruction for children in the preschool years.

There is good reason to believe that low-income preschool children respond well to sound math instruction. For example, the Building Blocks system (12, 88), a research-based curriculum that encourages teachers to formatively assess students during the course of instruction, found substantial positive effects on preschoolers’ math outcomes, compared to control groups (12–15). Furthermore, despite concerns among some educators that emphasis on math instruction could impede development in other areas such as language and literacy (89), the Building Blocks intervention positively influenced certain oral language skills (e.g., use of complex utterances) and had no impact on other oral language and literacy skills (e.g., letter recognition) (90). Thus, there is evidence that this intervention benefits math learning, does not negatively affect language and literacy learning, and may even benefit aspects of oral language learning. In our study, most classrooms used a respected curriculum that includes a math component and curricular-based assessments. The design of our study enables us to assess the added value of a regime of frequent, structured, direct assessments and tailored instruction.

## The Present Study

The present study builds on the previous literature by testing the impact of an iterative system of assessment and instructional support in preschool classrooms. We conducted a randomized trial to ascertain the impact of this system of assessment and instruction on student learning outcomes. Preschool classrooms serving predominately low-income children were organized into blocks, based on school and classroom characteristics, and randomly assigned within each block to either an intervention or a control condition. Control group classrooms varied in the intensity of math instruction, as detailed in *Methods*. Each teacher in the intervention condition assessed a randomly selected subset of students three times during the school year. After each round of assessment, teachers received feedback on each assessed student’s current proficiency and growth trajectory on each of the 12 skills (listed and described in Table 1), along with suggested targeted instructional strategies. At the end of the academic year, research staff assessed intervention and control students’ numerical, spatial, verbal, and literacy learning outcomes using standardized measures that were all independent of the Getting on Track (GoT) system.

Our main hypothesis was that students in intervention classrooms would show significantly better scores on standardized numerical and spatial measures at the end of the school year, compared to students in control classrooms. Additionally, we were both practically and theoretically motivated to test whether the intervention would impact verbal comprehension and print literacy skills. From a practical standpoint, some educators have expressed concern that increasing attention to math would take away instruction time from language and literacy and negatively impact children’s learning in those areas (89). From a theoretical standpoint, we turned to previously reported predictive and causal links between math skills on the one hand and verbal and literacy skills on the other hand (8, 90, 91). We did not have a specific prediction about the effect of our intervention on print literacy skills. However, we considered two possible mechanisms through which we might expect our math intervention to influence verbal comprehension skills. First, our intervention emphasizes math vocabulary, including words referring to measurement (e.g., “farthest”), quantity (“most”), shapes and shape features (“triangle,” “curve”), and spatial relationships (“inside,” “over”). Much of this vocabulary is used in a variety of contexts, and therefore we reasoned that acquiring this vocabulary could support the development of verbal comprehension more generally. Second, analogical reasoning about spatial relationships is another skill that is targeted in the intervention, and we predicted that strengthening children’s analogical reasoning in the spatial domain would also broadly support their verbal comprehension skills, which include analogical reasoning. Consistent with these ideas is the finding from experimental work that introducing preschoolers to spatial–relational vocabulary helps their performance on an abstract reasoning task (92).

## Results

To test the effect of a classroom-level intervention on individual student outcomes, and to account for dependence of individual students nested within classrooms, we estimated a two-level hierarchical linear model (93). Because we stratified classrooms into 23 blocks and randomly assigned classrooms within each block, our model includes fixed block effects.

### Impact on Student Math Outcomes.

Our main hypothesis was that the intervention would positively affect student numerical and spatial skills at posttest. To test this hypothesis with respect to numerical skill, we estimated the model

$$Y_{ij}=\gamma_{0}+\gamma_{1}I_{j}+\gamma_{2}A_{2ij}+\overset{22}{\underset{k=1}{\sum }}\beta_{k}B_{jk}+u_{0j}+u_{1j}A_{2ij}+e_{ij},$$ [1]

where $Y_{ij}$ is the numerical skill of child *i* in classroom *j* postintervention; $\gamma_{0}$ is the model intercept; $I_{j}=1$ if classroom *j* was assigned to the intervention condition, $I_{j}=0$ if not; $\gamma_{1}$is the average impact on *Y* of assignment to the treatment; $B_{jk}$ for *k* = 1, ... ,22 are indicators of the block membership of classroom *j* so that $\beta_{k}$ is the fixed effect of block *k*; $A_{2ij}$ is child age at posttest, centered around the grand mean; $u_{0j}$ is a zero-mean normally distributed random classroom effect; $u_{1j}$ is a zero-mean classroom-specific random increment to the age coefficient effect; and $e_{ij}$ is a normally distributed zero-mean random child effect. We assume $u_{0j}$ and $u_{1j}$ are bivariate normal in distribution with variances $\tau_{00}$ and $\tau_{11},$ respectively, and covariance $\tau_{01}.$ To assess the impact of the intervention on spatial skills, verbal comprehension, and literacy, we estimated the model shown in Eq. 1, except that $Y_{ij}$ is the appropriate outcome variable measured postintervention.

Model estimates are shown in Table 2. For numerical skills, we found a positive average intervention effect of ${\hat{\gamma }}_{1}=5.00,$ SE = 1.65, equivalent to a standardized effect size of 0.29.^†^ For spatial skills, we did not find a statistically significant effect of the intervention, ${\hat{\gamma }}_{1}=0.23,$ SE = 0.19, equivalent to a standardized effect size of 0.08.^‡^ To more clearly interpret the impact of the intervention on numerical skills, we can consider the relation between student age and numerical skills (Table 2). We see that the estimated difference of 5.00 points in students’ numerical skills between the intervention and control classrooms represents nearly 40% of the 12.61-point gain we would expect to see with an increase of 1 y in student age. We also considered the intervention impact in the context of the achievement gap, defined as the difference between the estimated average scores of children who are and are not growing up in poverty, based on analysis of the ECLS-K math assessment data (*SI Appendix*). In this context, the intervention impact narrows the estimated achievement gap in numerical skills by roughly 45%.

**Table 2.** Estimates of intervention impact, with and without pretest vocabulary as a covariate, on posttest math, literacy, and verbal skills

	Without pretest covariate			With pretest covariate		
	Coefficient	SE	T ratio	Coefficient	SE	T ratio
Numerical skills						
Treatment	5.00	1.63	3.07	5.00	1.37	3.65
Age	12.61	1.56	8.08	8.19	1.48	5.55
Pretest vocabulary				0.65	0.07	9.06
Spatial skills						
Treatment	0.230	0.328	0.702	0.201	0.323	0.624
Age	2.195	0.392	5.606	1.897	0.392	4.832
Pretest vocabulary				0.049	0.017	2.906
Verbal comprehension						
Treatment	4.00	1.35	2.97	3.82	1.11	3.45
Age	7.54	1.26	5.98	3.57	1.19	3.00
Pretest vocabulary				0.55	0.06	8.89
Literacy						
Treatment	1.71	3.56	0.48	1.20	2.99	0.40
Age	12.92	3.07	4.19	6.24	2.95	2.11
Pretest vocabulary				1.01	0.19	5.39

Numerical skills were measured by WJ-III Tests of Achievement Math Reasoning Cluster W scores. Spatial skills were measured by WPPSI-IV Block Design raw scores. Verbal Skills were measured by WJ-III Tests of Cognitive Ability Verbal Comprehension W scores. Literacy Skills were measured by WJ-III Tests of Achievement Letter–Word Identification W scores. Estimates for randomization block indicator variables (*γ*_01_ to *γ*_022_) are not shown. Pretest vocabulary was WJ-III Picture Vocabulary W scores.

### Impact on Student Verbal and Literacy Outcomes.

A second research question was whether the intervention influenced preschoolers’ verbal comprehension and literacy skills. To address this question with respect to children’s verbal comprehension skills, we ran the model in Eq. 1, with $Y_{ij}$ representing children’s posttest verbal comprehension scores. Results (Table 2) showed a positive effect of the intervention on posttest verbal comprehension skill, ${\hat{\gamma }}_{1}=4.00,$ SE = 1.35, equivalent to 0.29 in SD units.^§^ Considering this effect in the context of the relation between student age and verbal comprehension skills (Table 2), the intervention resulted in an increase in scores equivalent to roughly 48% of the gain in verbal comprehension skills associated with a 1-y increase in student age. To test the effect of the intervention on print-related literacy skills, we estimated the model shown in Eq. 1, with $Y_{ij}$ representing posttest literacy scores. The intervention effect in this model was nonsignificant, ${\hat{\gamma }}_{1}=1.71,$ SE = 3.56, equivalent to a standardized effect size of 0.04.^§^

Inclusion of pretested vocabulary as an additional covariate in these analyses produced nearly identical results (Table 2) except that the SE-estimated treatment effects were noticeably smaller. To control for multiple-hypothesis testing, we applied the Benjamini–Hochberg (96) procedure for controlling false discovery rate. Specifically, we rank ordered the four *P* values from *i* = 1 to 4 where rank 1 was assigned to the smallest *P* value (*P* = 0.005 for numerical skill to *P* = 0.635 for literacy). We chose q* = 0.05 as the adjusted critical value. Application of this procedure suggested rejection of the null hypotheses for numerical skill and verbal reasoning, but not for spatial skill or print-related literacy skill, indicating significant positive effects of the intervention on numerical and verbal reasoning skills but not on spatial or print-related literacy skills (see *SI Appendix* for details).

## Discussion

At entry to preschool, children vary substantially in their early math skills and these skills are correlated with children’s social and ethnic backgrounds. To promote learning in every child, we designed a regime of iterative assessment and instruction. Each assessment provided teachers with information about a child’s growth trajectory on a set of foundational skills, information designed to help teachers evaluate their students’ progress, reflect on past instruction, and strategize for the next phase of instruction. We evaluated this regime in a sample of 350 predominately low-income minority children. Children in classrooms of 24 trained teachers scored 0.29 SD higher on numerical skills at posttest than children in 25 randomly assigned control classrooms (*P* = 0.005). This impact on students’ numerical skills is equivalent to a 45% reduction in the estimated achievement gap between higher-income and lower-income students. Counter to our expectations, we did not see an intervention impact on students’ spatial skills.

Given that many math skills involve solving word problems and using analogical reasoning and vocabulary, a secondary aim of the study was to assess the impact of the regime on verbal comprehension and print-related literacy skills. We found a statistically significant and quite substantial effect on verbal comprehension, but no effect on print-related literacy skills.

### Explaining the Numerical vs. Spatial Math Results.

By individually assessing each child, we reasoned that a teacher would gain a clear understanding of what the key skills are, what each child knows, and what each child needs to know to advance to the next level. The typical progression of skills in the numerical domain is quite clear. Children typically learn the names and order of the small number words before knowing the meaning of those words; they learn how to determine the size of a set before mastering the cardinality principle; and they must master that principle before being able to order sets or compute operations on sets (52–56, 62). Once teachers understand and envision this developmental progression, they have crucial information that is needed to devise instructional activities and strategies relevant to each stage of numerical knowing. In contrast, the spatial skills as we defined them, and as studied in the cited literature, are less clear in the progression of their development. The process of tailoring instruction to spatial skill may therefore be less obvious than tailoring instruction to numerical skill. These sources of uncertainty may help us understand why teachers were able to use the assessments of numerical skill to better effect than the assessments of spatial skill. However, it is also possible that our spatial outcome measure, the Wechsler Preschool and Primary Scale of Intelligence (WPPSI)-IV Block Design subtest, was not sensitive to the particular spatial skills that students in intervention classrooms might have gained. Clearly, more research is needed on teaching and assessing spatial skill.

### Alternative Explanations for the Impact on Numerical Skills.

Do the observed results with respect to numerical skill reflect actual math learning or do they reflect test preparation? If the results do reflect actual math learning, is it reasonable to attribute the observed effects to the mechanisms suggested by the theory underlying the intervention?

#### Test practice.

One might reason that frequent assessment of math skills gave children experience and practice with test taking, which, in turn, gave them an advantage on the Woodcock–Johnson tests that we used as outcomes. However, in designing the study, we took care to avoid this possibility and therefore believe that this explanation is unlikely. First, we chose the Woodcock–Johnson Tests of Achievement-III (WJ-III) Applied Problems and Quantitative Concepts (94) subtests as our posttest measure of children’s numerical skills because it is a standardized, independently developed and validated instrument that taps the knowledge and skills we assess, but uses a different format and item content than we used on the GoT assessment. In *SI Appendix*, we explain in detail how the item formats and contents of WJ-III differ from those used in our assessment. Second, posttest measures were administered by trained assessors unaware of the experimental condition of the classroom. Neither researchers nor classroom teachers ever saw or interacted with the posttest measure. We therefore conclude that the gains reported here reflect real gains in skill and knowledge and not gains made possible by familiarity with item formats and test administration.

#### Emphasis on math.

Any intervention that supports the efforts of preschool teachers to encourage math learning could conceivably produce a substantial effect on math scores. This possibility is most likely to be true if math learning was not an aim of preschool educators in our control group (46, 48). However, this was not the case. A total of 42 of 49 participating classrooms (21 of the 25 control classrooms) reported using a math curriculum. In fact, two of our three sites were Head Start programs that require a math curriculum.

#### Individualized attention in math.

One might reason that our intervention generated an effect simply by increasing individual attention to children’s learning rather than by enacting a specific regime of assessment and instruction. This would be particularly plausible if control teachers devoted little individualized attention to children’s learning. However, 36 Head Start classrooms were required to use the Teaching Strategies’ GOLD observational assessment system. This system provides teachers with guidance on math instruction based on their ratings of children’s progress on four math objectives three times per school year. Significant, individualized, attention to children’s math learning was therefore a key component of math instruction even in the control classrooms in our study. To examine the possibility that the intervention effect reported here could be explained by a general increase in individualized attention to children’s math learning, we tested whether the effect was different for classrooms that did not regularly monitor their students’ math learning using the GOLD assessment and for classrooms that did use this system. We found no evidence of a differential effect (estimated effect difference = −0.259, *t* (24) = −0.070, *P* = 0.945); our estimate of the impact of the intervention in sites that did use the GOLD assessment was 5.07, *t* (24) = 2.60, *P* = 0.016, very similar to the 5.00 estimated overall average impact. We therefore conclude that the effect of the intervention reported here cannot be explained by simply putting math learning on the agenda or even by encouraging regular individualized assessment of children’s skills.

These results raise the question of why using our assessment–instruction regimen added value beyond the value contributed by the administration of the GOLD assessment system. Using GOLD, a teacher takes notes on a child’s behavior during interactions around math content and completes a rating scale assessing the child’s specific skills. The types of tasks that teachers observe to complete the scale on a particular skill vary widely, which may make it difficult to pinpoint skill level in an objective manner. Teachers may therefore, at times, rely on global impressions of a child’s skills when they complete GOLD ratings. In contrast, our assessment requires teachers to administer the same task to all children using a standardized protocol. Teachers either report a child’s specific response or use specific guidelines to decide whether a child is correct based on the child’s response. We hypothesize that a key element that makes our system effective is that teachers witness the behaviors that children of different skill levels exhibit as they grapple with the same assessment tasks, giving teachers a better understanding of the skill trajectory and a more objective assessment of each child’s current skill level along that trajectory than they might achieve with GOLD ratings. Teachers may find it difficult to detect errors in children’s thinking by observing their ordinary interactions with children. For example, preschool children often learn to say the words used in counting before they understand the numerical concepts that those words express. Hence, children’s counting performance may suggest the false impression that they understand mathematical concepts that in fact they lack. By heightening teachers’ awareness of the gaps in their students’ understanding, the intervention appears to have enabled teachers to target their instruction more effectively.

### Explaining Findings in the Verbal Domain.

We also saw a positive intervention effect on students’ verbal comprehension skills. This effect is not surprising given the relationship between math and language comprehension reported in the literature and the heavy emphasis on mathematical vocabulary and spatial analogical reasoning in the intervention. Conversely, we found no difference between the intervention and control conditions on print literacy. Taken together, these findings are generally consistent with Sarama et al.’s (90) findings that a year-long pre-K math intervention improved some oral language skills (e.g., complex utterances; retelling a story with minimal prompting), but not others (e.g., sentence length), and had no effect on letter recognition skills. Although some studies have shown concurrent and predictive relations between math achievement and print literacy skills, including letter–word identification (91, 97, 98), we had no expectations that a 1-y math intervention would positively influence skill in identifying words and letters in preschool children.

## Conclusion

Following Bailey et al. (19, 99), we do not assert that longitudinal adaptive assessment and instruction as we have conceived them are sufficient to eliminate, or even substantially reduce, social inequality in math learning over the life course. Rather, we regard our results as suggesting that this kind of iterative regime has potential as a first important step in overcoming inequality and that this approach can begin early and have positive effects. We are encouraged by successful interventions of this type, reviewed above, at the elementary level. Given these findings, we think there is good reason to expect that a coherent dynamic regimen of assessment and instruction, implemented across the course of early and middle childhood, could substantially increase math learning and reduce inequality linked to children’s demographic origins.

## Methods

We provide here a brief summary of the development of the longitudinally adaptive assessment system, followed by a description of the methods used in the randomized trial. For details about the methods and procedures underlying the development of this regime, refer to *SI Appendix*.

### Development of the Regime.

The GoT assessment contains game-like tasks measuring 12 skills across two domains: numerical thinking (cardinality, counting, written numeral knowledge, comparing and ordering sets, and operations), and spatial thinking (mental rotation, shape composition, shape features, shape knowledge, patterns and structure, spatial relationships, and mathematical vocabulary). Each skill is measured by one or more game-like tasks, described in Table 1.

To design an assessment that would measure these 12 skills, our research team and a team of collaborating practitioners constructed assessment items by adapting tasks established in the literature and by developing additional tasks when there were no suitable tasks reported in the literature. We then conducted a field test to refine our item pool and assess the reliability and validity of the assessment. We next addressed a key logistical challenge—minimizing assessment administration time while maximizing information about each child’s current proficiency—by engineering short longitudinally adaptive assessments in the form of booklets. Specifically, we designed eight assessment booklets (four for spatial thinking and four for numerical thinking) that contained between 10 and 22 assessment items and that varied in difficulty. We then created an item-response model for estimating each child’s current proficiency, taking into account the child’s performance on the most recent and prior assessment administrations, as well as age. Under this model, we were able to assign each child to a booklet of appropriate difficulty based on our best estimate of the child’s current skill level. This design increased efficiency by allowing teachers to administer only test items that are in the neighborhood of the child’s skill level (100, 101), rather than administering all test items at each round of assessment.

Following our construction of the assessment, we developed empirically based targets for student proficiency, designed visual and textual feedback to help teachers interpret assessment data, and developed suggested instructional strategies to help teachers tailor instruction and move students along learning trajectories (50, 65, 102). Further detail about the development of the assessment system, instructional strategies, and the model for estimating student proficiency is provided in *SI Appendix*.

### The Randomized Trial.

#### Recruitment and participants.

Five organizations that offer instruction for children ages 3 to 5 y in and around a large Midwestern city were invited to participate. Three organizations agreed: one system of parochial elementary schools and two nonprofit organizations that run Head Start programs in rural, suburban, and urban areas. The Head Start organizations served students from low-income families (100% of students were eligible for free/reduced-price lunch), as is a prerequisite for Head Start. The parochial schools served a somewhat broader range of income levels, with an average of 46% of students eligible for free/reduced-price lunch (range 3 to 92%).

Within the two Head Start organizations, we invited all classrooms taught by at least two teachers to participate, as was requested by the organizations’ administration. Within these organizations, teachers in 36 of 51 classrooms agreed. Within the parochial school organization, administrators invited all preschool teachers in 32 schools to participate, and teachers in 10 schools (13 classrooms) agreed. Across all 49 participating classrooms, 88 teachers participated either as the single lead teacher in their classroom or as teacher teams, depending on the requests of their organizations’ administrators. The 13 parochial school classrooms were full-day programs. Of the Head Start classrooms, 21 were full-day programs (6 h, 5 d/wk) and 15 were half-day programs (3 h, 4 d/wk). Across the three organizations, teachers had an average of 13 y of teaching experience (SD = 9 y), with an average of 9 y (SD = 7) among teachers in the parochial schools, 10 y (SD = 9) among teachers in one of the Head Start organizations, and 12 y (SD = 9) among teachers in the other Head Start organization.

Parents of all students from participating classrooms were sent forms requesting consent for their child’s participation. We received consent for 747 of 1,010 students. Among students with consent, teacher-reported racial composition was as follows: 45% Black/African-American, 27% Hispanic, 15% non-Hispanic White, 8% biracial/multiracial, 4% Asian, and 1% other/not listed. Fifty-one percent of students with consent were male. Following the recommended sample size of an a priori power analysis, we randomly selected 8 consenting students in each classroom to be pretested and posttested, excluding students with individualized education plans or who spoke too little English to understand assessment instructions. The final sample included 350 students.

#### Treatment assignment.

Within each of the three organizations, we created randomization blocks by matching classrooms on key variables and then randomly assigned classrooms within each block. Within each of the Head Start organizations, we matched classrooms within schools and, when possible, according to classroom schedule (e.g., full day or part day). When multiple classrooms from the same school had the same schedule (e.g., four full-day classrooms within the same school), we matched them at random. Administrators in the parochial schools preferred not to randomize classrooms within schools. Therefore, we matched schools on the following factors, in order of priority: number of preschool classrooms, percentage of school population qualifying for free/reduced-price lunch, and racial/ethnic composition. Only two parochial schools had more than one participating classroom, and we matched these two schools to keep the groups balanced, resulting in a randomization block of five classrooms. Taken together, this procedure resulted in a total of 23 randomization blocks, of which 22 were pairs of classrooms and one was a block of 5 classrooms.

Within each of the 23 blocks, one school/classroom was randomly assigned to the intervention group and the other was assigned to the control group. Within the block of five classrooms, two classrooms were assigned at random to the intervention condition and three to the control condition. In total, 24 classrooms were assigned to the intervention, and 25 were assigned to the control. Following random assignment of classrooms to treatment conditions, we found that intervention and control groups were statistically balanced on all pretreatment variables including demographic variables, full-day versus half-day kindergarten, probability of obtaining parental consent, and pretested vocabulary (see *SI Appendix* for details).

#### Intervention procedures.

Prior to assessing any students, teachers attended a workshop where they learned about the skills on the assessment, practiced administering several of the tasks, and obtained online access to a small bank of lessons and resources related to the assessed skills. During the school year, after each round of assessment, teachers participated in half-day workshops (three in total) that enabled them to reflect on their experience with administering the assessment and on their observations of students’ responses, interpret the data visualizations provided on the website, and collaborate to plan differentiated instruction. Drawing from recommended lessons on the website, teachers collaborated to plan differentiated instruction. The workshops also trained them to administer new tasks appearing at each subsequent assessment period.

We did not expect experimental teachers to assess all of their students, but rather, randomly assigned each experimental teacher a number of students to assess, depending on the number of teachers per classroom. In classrooms with two teachers, each teacher was assigned 6 students, totaling 12 students per classroom; classrooms with one teacher were assigned 8 students. Teachers were free to assess more students than assigned. Teachers assessed their assigned students three times over the year. For each round of assessment, a GoT team member observed and coached teachers as they administered the assessment to one or two of their students, providing support and feedback. Three teachers demonstrated considerable difficulty administering the assessment (exhibiting numerous mistakes that could affect the child’s responses). In those cases, the GoT member provided one more coaching session. These teachers then went on to assess the rest of their assigned students without the coach. Additional interactions between GoT team members and teachers were as follows: 1) reminder emails to teachers to complete their assessments and attend upcoming coaching sessions and workshops, 2) follow-up emails after each coaching session that summarized the session and outlined next steps, and 3) periodic emails inviting teachers to email coaches with any questions about the assessment or materials.

Feedback was a key component of the intervention. After administering each assessment, the teacher entered the student responses into a website application, which computed each student’s score on each skill (see *SI Appendix* for details). We then used these scores to provide visual and textual feedback to teachers about each student’s proficiency (see *SI Appendix* for an example). For teachers to be able to accurately interpret the visual feedback, it was important for them to understand that each student’s proficiency was measured with uncertainty, represented as a symmetric 68% confidence interval.

#### Compliance.

We measured compliance in terms of the fraction of students each teacher team assessed out of the students they were assigned and in terms of the frequency with which teacher teams assessed each assigned student. Of the 24 teacher teams in the experimental classrooms, 22 of 24 assessed all of the students assigned to be assessed at least once. In the other two classrooms, 75 and 92% of the assigned students were assessed at least once. In terms of frequency, 70% of assigned students were assessed three times, 17% were assessed two times, 11% were assessed one time, and 2% were never assessed. In 11 classrooms, the number of students assessed exceeded the number assigned, as teachers had the option of assessing more than the assigned number.

#### Regular math curriculum and instruction.

Regular math instruction varied between the parochial schools organization and the two Head Start organizations. In compliance with the Head Start requirement for research-based early childhood curricula, the teachers of all 36 Head Start classrooms used *The Creative Curriculum for Preschool*, which includes learning objectives in numerical and spatial math, as well as other domains. Head Start also requires ongoing monitoring of student outcomes, and thus, all Head Start teachers also used Teaching Strategies’ GOLD, an observational assessment of student learning that is aligned with the learning objectives of *The Creative Curriculum*. Using GOLD, teachers completed observational ratings of each of their students’ proficiency in math and other domains three times over the course of the school year. Additionally, teachers of 22 classrooms (those in one of the Head Start organizations) had access to supplemental math lessons in *Hand2Mind*.

Among the 13 teachers in the parochial schools, 3 used *Everyday Math*, 1 used *Houghton-Mifflin Pre-K Math*, 1 used *The Creative Curriculum for Preschool*, 1 used *Big Book Math*, and 7 used no curriculum. Unlike the Head Start classrooms, all of which used an observational math assessment system, none of the parochial school classrooms used a math assessment system.

In all three organizations, the proportion of classrooms that used a math curriculum was similar across teachers assigned to the intervention (21 of 24 classrooms) and control conditions (21 of 25 classrooms). Teachers in both conditions continued to use their curricula during the trial.

#### Pretest and posttest procedures and measures.

Research staff blind to experimental condition administered individualized pre- and posttests in a quiet location in each child’s school. At pretest, we measured vocabulary and, at posttest, we measured numerical and spatial skills––our math outcomes of interest––as well as verbal comprehension and literacy skills. The pretest measure was administered during single 5- to 10-min sessions, and posttest measures were administered in two 15- to 20-min sessions, split across 2 d. Subtests from three normed, standardized assessments were used to measure the outcome variables: the WJ-III Tests of Achievement (for vocabulary, numerical, and print-related literacy skills), the WJ-III Tests of Cognitive Abilities (for verbal comprehension), and the WPPSI-IV (for spatial skill). Outcomes measured using WJ-III subtests were W scores, which are transformed Rasch ability scales, and were calculated using Compuscore 3.1. The spatial outcome, measured using the WPPSI-IV, was raw scores.

#### Pretest vocabulary skills.

The WJ-III Tests of Achievement Picture Vocabulary subtest requires children to provide the name for pictures, measuring their expressive vocabulary for single-word items. Split-half coefficients range from 0.76 to 0.84 for children ages 3 to 5 y (94).

#### Posttest numerical skills.

We administered the WJ-III Applied Problems Math Reasoning Cluster, consisting of the Quantitative Concepts and Applied Problems subtests, to measure numerical skills. Quantitative Concepts measures children’s knowledge of basic concepts and procedures, including measures of counting, comparing, and ordering sets, and mental arithmetic. Applied Problems measures children’s ability to apply math procedures and concepts to real world problems. Most questions on these subtests involve numerical math, with a small subset of items tapping spatial aspects of mathematical knowledge, such as identifying shapes and knowledge of spatial vocabulary (e.g., “largest,” “smallest”). Split-half reliability coefficients for children ages 3 to 5 y range from 0.86 to 0.93 on Quantitative Concepts and from 0.92 to 0.94 on Applied Problems (94).

#### Posttest spatial skills.

To capture spatial skills, we administered the WPPSI-IV Block Design subtest to measure spatial skill. This subtest assesses children’s facility at recreating a design using blocks, incorporating awareness of shapes and position, as well as the ability to rotate and manipulate blocks. Split-half reliability coefficients for this test range from 0.81 to 0.84 for children ages 3 to 5 y (95).

#### Posttest verbal comprehension skills.

We assessed students’ verbal comprehension skills using the WJ-III Tests of Cognitive Ability Verbal Comprehension subtest, which measures knowledge of synonyms and antonyms, reasoning about verbal analogies, and skill in identifying objects. Split-half reliability coefficients range from 0.88 to 0.89 (94).

#### Posttest print literacy skills.

We measured literacy skills with the WJ-III Letter–Word ID subtest, which assesses children’s ability to identify printed letters and words. For children ages 3 to 5 y, split-half reliability coefficients range from 0.97 to 0.99 (94).

#### Human subjects review.

This study was approved by the University of Chicago Institutional Review Board (IRB) under IRB14-0845 on 4 March 2015. The team was approved to help teachers use GoT assessment booklets in their classrooms, to administer standardized assessments to a subset of the teachers’ students, and to collect survey data from teachers.

## Supplementary Material

Supplementary File

## Data Availability

Some study data are available upon request.

## Footnotes

^†^Standardized to 5-y-old children in the WJ-III norming sample (94).

^‡^Standardized to 5-y-old children in the WPPSI-IV norming sample (95).

^§^Standardized to 5-y-old children in the WJ-III norming sample (94).
