# Supplementary material for: Longitudinally adaptive assessment and instruction increase numerical skills of preschool children
Source: Proc Natl Acad Sci U S A. 2020 Oct 26;117(45):27945–53. doi: 10.1073/pnas.2002883117 (PMC7668039; doi:10.1073/pnas.2002883117)
Supplement: Supplementary File [file pnas.2002883117.sapp.pdf]

Supporting Information

For

Longitudinally Adaptive Assessment and Instruction Increase Numerical Skills of Preschool Children

Stephen W. Raudenbush[1], Marc Hernandez[2], Susan Goldin-Meadow[3], Cristina Carrazza[3], Alana Foley[3], Debbie Leslie[4], Janet E. Sorkin[3], & Susan C. Levine[3]

I. Development of the Longitudinal Adaptive Assessment and Instruction ("LAAI") System

We developed the assessment in three phases: (1) constructing tasks and items; (2) field testing and refining the tasks; and (3) engineering booklets that are adapted to children's current skill. Following our construction of the assessment, we developed empirically-based targets for student proficiency, designed visual and text feedback to help teachers interpret assessment data, and developed suggested instructional strategies that teachers could use to tailor instruction to move students along established learning trajectories (e.g., (52, 99, 100)).

Task and item construction. Our team of researchers and practitioners developed the content of the assessment by adapting tasks that were established in the literature, such as the Give-a-Number task (55, 56) as well as by developing tasks for skills for which there were not suitable tasks in the literature. We strove to create tasks that are engaging for children, easy and efficient for teachers to administer, informative for teachers (e.g., revealing strategies or mistakes that children make), and instructionally relevant – that is, amenable to influence by instructional activities that can be implemented in the classroom. One of our goals was to assess skills using

tasks that required verbal responses as well as tasks that were less verbally demanding, to assess whether children had a concept but lacked the verbal skills to express this knowledge.

Field test and analysis of field test data. We field tested 320 items from 24 methodologically distinct tasks with 400 students aged 3-5. Our analyses of student response data from the field test informed the final set of items in our item pool and allowed us to examine the reliability and validity of the assessment. First, we evaluated construct validity by testing *a priori* hypotheses about whether certain tasks or combinations of tasks were measuring certain skills, while also examining reliability. Specifically, using item response theory (101), we fit 2-parameter logistic (2PL) IRT models to the field test data and examined estimated item discriminations and difficulties. If items on a task or set of tasks hypothesized to measure a certain skill displayed similarly high discrimination parameters but a range of difficulty levels, this would provide evidence of reliable and valid measurement. These analyses suggested that there were 12 distinct skills being measured by 22 tasks. Additionally, we calculated Cronbach's alphas for each hypothesized skill (from one or more tasks) to examine the internal consistency, and examined item-test correlations to see how well individual items related to the skill overall. In a few cases, we discarded items that did not fit the skill they were intended to measure and that undermined reliability.

Next, we tested hypotheses about which skills were driven by latent, domain-level proficiencies in numerical and spatial thinking using CFA. We found the strongest support for a two-factor solution, where 5 skills loaded more strongly onto a hypothesized numerical thinking factor, while the remaining 7 skills loaded more strongly onto a hypothesized spatial thinking factor, including 2 skills (Patterns and Mathematical Vocabulary) that we viewed as spanning numerical and spatial domains. The factor scores from each of these domains were strongly

correlated, $r = .78$. These findings are consistent with those of Mix and colleagues (66, 67), who also found evidence for spatial and numerical factors that were separate but highly correlated in kindergarten, 3rd and 6th graders. To evaluate convergent validity of the numerical and spatial scales that emerged from the factor analysis, we examined bivariate correlations between each scale and independent, standardized measures of numerical and spatial math skill. To capture numerical math skill, we used scores from the WJ-III Tests of Achievement Math Reasoning Cluster (which comprises two subtests – Quantitative Concepts and Applied Problems, which mainly tap numerical skills), and to capture spatial math, we used a mean *z*-score composite of two measures – the Spatial Reasoning subtest from the WJ-III Tests of Cognitive Ability and the Block Design subtest from the WPPSI. We compared the magnitudes of the correlations by converting the correlations to *z*-scores using Fisher *r*-to-*z* transformations, computing the asymptotic covariance of these estimates, and then using these covariances in an asymptotic *z*-test (102). We found that the WJ-III Math Reasoning scores were more strongly correlated with the numerical factor ($r = .87$) than with the spatial factor ($r = .76$), $z = 3.52$, $p < .001$. In parallel, the spatial composite scores (from the WJ-III Spatial Reasoning and WPPSI Block Design) were more strongly correlated with the spatial factor ($r = .70$) than with the numerical factor ($r = .60$), $z = 1.72$, $p = .043$.

Engineering a Longitudinally Adaptive Assessment. During the field test, it took 120 minutes, over 8 sessions, to administer 320 items to a child. In contrast, based on discussions with teachers and local school administrators, we estimated that it would be impractical to spend more than 15 minutes to assess each child on each round of assessment. Hence, each round of assessment might entail 40-45 items. Is it possible to obtain instructionally useful information about 12 skills using only 45 items?

Our affirmative answer to this question is based on three facts predicted by theory and supported by our analysis of our field test data. First, the 12 skills we sought to measure load strongly on two factors: a numerical and a spatial factor. This means that information about one skill carries information about another. Using what is called “multivariate shrinkage” in the Bayesian Literature (90), we exploit information about all skills to obtain an estimate prediction of any one skill that is more accurate than would be possible based only on the items that measure each specific skill of interest. Through this approach, we were able to estimate a child’s proficiency on each of the 12 skills at each time point without having to directly measure each skill during each round of assessment.

Second, the assessment of a specific child’s skill can be remarkably efficient if the difficulty of the items used to assess that child’s skill are located in a small neighborhood around that child’s skill. This second fact is the basis for what is called “Computerized Adaptive Testing” (CAT; (103, 104)). After an examinee responds to an item administered by computer, CAT selects the next item to be maximally informative about that examinee’s skill. Specifically, for item response t, the examinee’s skill is estimated based on the number and difficulty of the t-1 items that the examinee had previously answered correctly. CAT minimizes the number of items required to obtain a maximum tolerable standard error of measurement. We could not use CAT because our assessment tasks involve manipulatives that the child can touch. However, we were able to develop a related approach that we call “Longitudinally Adaptive Assessment” (or LAA). Using this approach, we constructed adaptive assessment booklets that were maximally informative for leveled segments of the skill distribution (as described under “Adaptive assessment booklets”, below), and used all information from prior assessments to determine

which booklet the child would be administered at the next time of assessment. This increases the efficiency of each subsequent assessment.

Third, evidence accumulates across assessments according to a Bayesian learning model. Using this model, each additional assessment, a) enables us to obtain a new, improved estimate of the child's growth trajectory to date; b) uses all available information from the most recent and prior assessments to produce an estimate of the child's current proficiency in each of the 12 skills, including those not tested at a given point in time; c) gives evidence of change in each skill since the last assessment and therefore provides information to the teacher about how well the child responded to the most recent instructional plan; and d) supports a prediction of the child's proficiency in each skill at the next assessment time, which we use to design the next assessment.

Adaptive assessment booklets. Ideally, each child's assessment would be closely tailored to that child's current proficiency. However, in this early stage of our LAA system, we approximated the child skill distribution by devising three booklets – A, B, and C – that varied in difficulty. The evidence accumulated across the first t-1 assessments determined the choice of booklet at time t for t = 2,3. At time t = 1, all children were administered an initial booklet that covered a range of difficulty levels. For a given estimate of a child's current proficiency, IRT gives us an estimate of how much information would be supplied by each booklet, so we chose the most informative booklet each assessment round. However, at time t = 2, we set the constraint that the child could be assigned either booklet A or booklet B, but not booklet C, in order to pace the professional development required for teachers to learn how to administer the tasks in each booklet.

The test information curves for each booklet in the Numerical Thinking and Spatial Thinking domains are shown in Figure 1. We designated the cutoff for assignment into each booklet as the proficiency level at which the information curves for two adjacent booklets intersect. For example, as can be seen in Figure 1, the information curves for Numerical Thinking Booklet A and Booklet B intersect at the proficiency level of -1.19. Therefore, a student with a Numerical Thinking score of -1.25, for example, would be assigned to Booklet A, whereas a student with a score of -1.10 would be assigned to Booklet B.

At each time point, 14 estimates of child proficiency were generated – one for each of the 12 skills, as well as one for the domain of numerical thinking and another for the domain of spatial thinking. The skill-level estimates were provided to teachers to provide detailed information about the child's current mathematical knowledge, while the domain-level estimates were used for determining the appropriate booklet assignment for the subsequent assessment, as well as for providing estimates of students' proficiency on skills that were not tested at a specific time point.

Setting Targets for Student Growth. Given our overarching goal of narrowing the achievement gap in mathematical skill at kindergarten entry, we identified targets for each of the 12 skills for children at age of entry to kindergarten. These targets were empirically grounded in math achievement data gathered from a large, nationally representative sample of students tracked from Kindergarten through grade 8 in the Early Childhood Longitudinal Study - Kindergarten Cohort (ECLS-K). Using the ECLS-K data, we modeled growth in children's math achievement from kindergarten through 8th grade as a function of children's family income and racial/ethnic background. These models showed that White children from higher-income families scored, on average, at roughly the 60th percentile in math achievement in the fall of

kindergarten, while the average scores for non-White children (African American and/or of Hispanic descent) from lower-income families were at the 22nd percentile. This gap persisted through the fall of 8th grade.

Our aim was to put each of our predominately low-income children on a trajectory of growth displayed by higher-income White children in ECLS-K – that is, at the 60th percentile by age 5.5 years. We therefore administered the Woodcock-Johnson Tests of Achievement-III (WJ-III) Math Reasoning Cluster, a nationally normed standardized test similar to that used in ECLS-K. We assumed that our children who scored at the 60th percentile at age 5.5 of this test had similar math skills to 5.5-year-old ECLS students who scored at the 60th percentile on the ECLS test. Next, we assumed that 5.5-year-olds scoring at the 60th percentile on math globally would also score, on average, at the same percentile on the 12 constituent skills. Then, using our field test data, we estimated a family of growth curves for each skill. These were quadratic in age and provided an estimate of where a student at the 60th percentile at age 5.5 would have scored on each skill between ages 3 and 5.5. We discussed with teachers the aim of eliminating social disparities in math achievement at age 5.5 by helping children move toward the 60th percentile trajectory on each and every skill that, under our assumptions, constitute the global social disparity.

Designing Feedback and Instructional Support for Teachers. To support teachers' instruction in response to assessment of their students, we developed visual and textual feedback to illustrate and describe their students' growth along learning trajectories for each of the 12 skills, and provided lessons to support continued growth. We designed two types of graphics to provide teachers with visual feedback for each of the 12 skills (see Figure 2). The first (Figure

2a) displays the child's estimated location along a linear trajectory, within a 68% confidence interval, representing his or her level for that skill. The trajectory line features benchmarks indicating the tasks that a child can successfully accomplish and those that the child has not yet accomplished. For example, within the skill of Cardinality, there are 4 benchmarks, ordered from least to most advanced: "Knows 1", "Knows 2", "Knows 3", and "Knows the Cardinal Principle". A color-coded bar signifies the child's skill level relative to the estimated target for children of the same age. The second graphic displays the child's status for multiple assessment periods (also within a 68% confidence interval; Figure 2b), illustrating the child's growth over time. Estimates are superimposed over an image displaying the "target" trajectory curve (based on the 60th percentile), providing information as to whether the child is moving in the direction of our target for that skill at age 5.5. This graphic also is demarcated by benchmarks (see the vertical axis of Figure 2b).

For each skill, we also created textual feedback describing children's current levels and instructional next steps for ranges of scores, resulting in three to four leveled text blurbs for each skill. The textual feedback blurbs included information about the mathematical content that children currently know and the content they should next encounter in that skill area, as well as information about suggested pedagogical strategies to support children's learning. For example, for cardinality, one feedback blurb described the Current Level as follows: "These children can identify and make sets of objects for small numbers (1, 2, and 3). However, they may not yet be able to create larger sets that they cannot subitize (automatically "see")." For Next Steps, it suggested: "Encourage these children to make and count sets of greater than 3 objects. Help them connect the counting process to quantity by labeling the total as well as pointing and counting (e.g., You have *five* crackers on your plate. One, two, three, four, *five*"). Reinforce that the

number word represents the whole set by gesturing around all of the objects." The content information in these feedback blurbs (e.g., the first sentence in the example) was based on research on early mathematical learning trajectories (52, 99, 100). The pedagogical information in the blurbs was based on available research from cognitive science (e.g., (55, 56, 60, 105) as well as research on effective instructional practices (e.g., (34, 81–83, 106–109)). In some cases, the research basis for this textual feedback was rich and specific; in others, it was less robust. In these latter cases, we drew on the experience and expertise of our team composed of researchers and practitioners to develop the blurbs, often extrapolating from research in related areas and from best practices in the field.

Finally, drawing on the same research base that we used to develop the textual feedback blurbs, we created a bank of approximately 50 lessons with input from a collaborator team of expert teachers. During the RCT, *GoT* teachers were able to access the lessons from a *GoT* resource website, where they were organized by the *GoT* skill(s) they targeted and their level of difficulty within those skills. In addition to basic instructions for the activity, each lesson provided a list of the main *GoT* skills it emphasized, mathematical vocabulary to use when implementing the lessons, suggestions for adjustments to make it easier or more challenging, and ways for teachers to look for children's understanding of the skills and concepts while implementing the lesson. The lessons also embedded research-based pedagogical strategies, such as the use of gesture and comparison to present or reinforce concepts (34, 81–83, 106–109). With only 50 lessons, the lesson bank was not designed to be a full curriculum, but many teachers used it to supplement curricular materials they already used or to help them teach *GoT* concepts or skills that for which they did not have ideas or curricular materials.

II. The Randomized Trial

Testing Balance Between Treatment Conditions. Following random assignment of classrooms to treatment conditions, we checked balance between intervention and control groups on key variables at the school, classroom, and student levels. At the school level, we tested balance on the percentage of students who qualified for free or reduced-price lunch in the parochial schools only (100% of students qualified for free or reduced-price lunch in Head Start schools). At the classroom-level, we examined program schedule (part- or full-day) for Head Start schools only (all of the parochial school classrooms were full-day). At the student level, we first examined whether the rate of consent differed for treatment and control groups. Subsequently—including only students for whom we obtained parent consent—we examined gender, age, and pretest vocabulary. Results showed no significant differences between treatment groups on any variables.

Descriptions and results of models used to check balance between intervention and control classrooms are shown in Table 1. The first column of this table lists the pre-treatment variables on which we tested balance. The second and third columns indicate the statistical method used to test the difference between the intervention group and the control group on each of these variables. The fourth and fifth columns provide the intervention and control means on each variable. The sixth and seventh columns supply the test statistic and nominal p value for each test.

When testing balance on student characteristics, we included covariates in our models that represented classroom membership in the randomization blocks. When testing balance on continuous student variables (e.g., age), we used Hierarchical Linear Models (HLM) and when testing balance on dichotomous outcomes (e.g., whether the child's race/ethnicity was White),

we used Hierarchical Generalized Linear Models (HGLM), specifying a Bernoulli distribution. In the HLMs and HGLMs, student variables were modeled at Level 1, classroom variables were modeled at Level 2, and we allowed the Level-2 intercept to vary randomly between classrooms.

Difference Between *GoT* Assessment and Outcome Measures. In the Discussion section of the main text, we discuss our choice of the Woodcock-Johnson Tests of Achievement – III (WJ-III) Applied Problems and Quantitative Concepts subtests as our posttest measure of children's numerical skills. We chose these because they are independent, standardized instruments that measure the same knowledge and skills as the *GoT* assessment, but that differ from the *GoT* assessment tasks both in terms of item content and administration approach. Here, we provide some examples of these differences. In our task "Showing a Set with Fingers", designed to measure children's cardinal number knowledge, children are shown an image depicting a set of objects (e.g., three identical stars) and are asked to show how many objects there are using their fingers. In our other task measuring cardinal number knowledge, "Give a Number", children are asked to produce a set of a named quantity (e.g., "Can you give the penguin three fish?"). In neither of these tasks was a verbal response required. The test items on the WJ-III that tap cardinal number knowledge differ both in the stimuli presented to the child and in the format of the required response. For example, children are shown an image containing a set of familiar objects that differ from those used in the *GoT* assessment, and are asked "How many [objects] are there?". A verbal number-word response of is required in order for the response to be marked as correct by the assessor. In another item on the WJ-III that taps cardinal number knowledge, children are shown an image depicting a mixed set of familiar objects and are asked "How many [objects] are there?", referring to one type of object in the mixed set. Again, a verbal number word response is required. Thus, the stimuli shown to children differ, the

homogeneity of the objects in the sets differs (e.g., homogeneous on *GoT* vs. heterogenous on WJ), and the response format differs (non-verbal number gesture or producing a set on *GoT* vs. verbal number word on WJ). Additionally, in the second example, not only do the stimuli and response formats differ from those of the *GoT* test items, but, additionally, the task is a measure of knowledge transfer: children must apply their cardinal number knowledge to extract the quantity of one type of object from a larger quantity of mixed objects, rather than determining the cardinal value of the whole set. The above examples are in no way unique. We carefully reviewed each item on the WJ-III Applied Problems and Quantitative Concepts and found that test item formats and administration differed from that of the *GoT* assessment.

Controlling for Multiple Testing Bias. We collected data on four outcomes – numerical skill, spatial skill, verbal comprehension, and early literacy – and therefore tested four research hypotheses. To control for false discovery rate, we applied the Benjamini-Hochberg procedure. Specifically, we rank-ordered the four *p*-values from i=1 to 4 where rank 1 was assigned to the smallest *p* value (p=.005 for numerical skill to p=.635 for literacy). We chose q*=.05 as the adjusted critical value. Application of this procedure suggested rejection of the null hypotheses for numerical skill and verbal reasoning but not for spatial skill or literacy (see Table 2).

Table 1

Pre-intervention balance checking models, model estimates, and test statistics for school, classroom, and student characteristics

<table>
<tr><th></th><th>Model Type</th><th>Fixed & Random Effects in Model</th><th>Intervention</th><th>Control</th><th>t</th><th>p</th></tr>
<tr><td>School</td><td></td><td></td><td></td><td></td><td></td><td></td></tr>
<tr><td>% F/R Lunch</td><td>Independent-Samples T-Test</td><td>Experimental condition</td><td>39%</td><td>46%</td><td>-0.38</td><td>.712</td></tr>
<tr><td>Classroom</td><td></td><td></td><td></td><td></td><td></td><td></td></tr>
<tr><td>Schedule: Full-Day</td><td>Chi-square Test of Association</td><td>Experimental condition</td><td>56%</td><td>61%</td><td>0.11§</td><td>.735</td></tr>
<tr><td>Student</td><td></td><td></td><td></td><td></td><td></td><td></td></tr>
<tr><td>Parent Consent</td><td rowspan="2">Hierarchical Generalized Linear Model (Bernoulli Distribution)</td><td rowspan="3">Fixed: Classroom randomization block & experimental condition (Level 2)

Random: Level-2 intercept</td><td>79%</td><td>78%</td><td>0.15</td><td>.886</td></tr>
<tr><td>Gender: Male</td><td>51%</td><td>49%</td><td>0.48</td><td>.634</td></tr>
<tr><td>Age (Years)</td><td rowspan="2">Hierarchical Linear Model</td><td>4.27</td><td>4.36</td><td>-1.01</td><td>.321</td></tr>
<tr><td>Vocabulary (W Score)</td><td>Fixed: Student age (Level 1), classroom randomization block, and experimental condition (Level 2)

Random: Level-2 intercept & level-1 slope associated with student age</td><td>460.53</td><td>460.22</td><td>0.22</td><td>.830</td></tr>
</table>

Note. % F/R Lunch = Percentage of students in school eligible for free or reduced-price lunch. Home language = primary language spoken in child’s home. F/R lunch is reported at the school level for parochial schools only; 100% of students in Head Start schools received F/R lunch. Classroom schedule is reported for Head Start schools only; all classrooms in parochial schools followed a full-day schedule. § Statistic for chi-square test of association is Pearson χ^2

Table 2. Results of Benjamini-Hochberg procedure for controlling false discovery rate

**Outcome**	***p***	**rank (*i*)**	**(i/4)q***
Numerical	0.005	1	0.0125
Verbal	0.006	2	0.025
Spatial	0.241	3	0.0375
Literacy	0.635	4	0.05

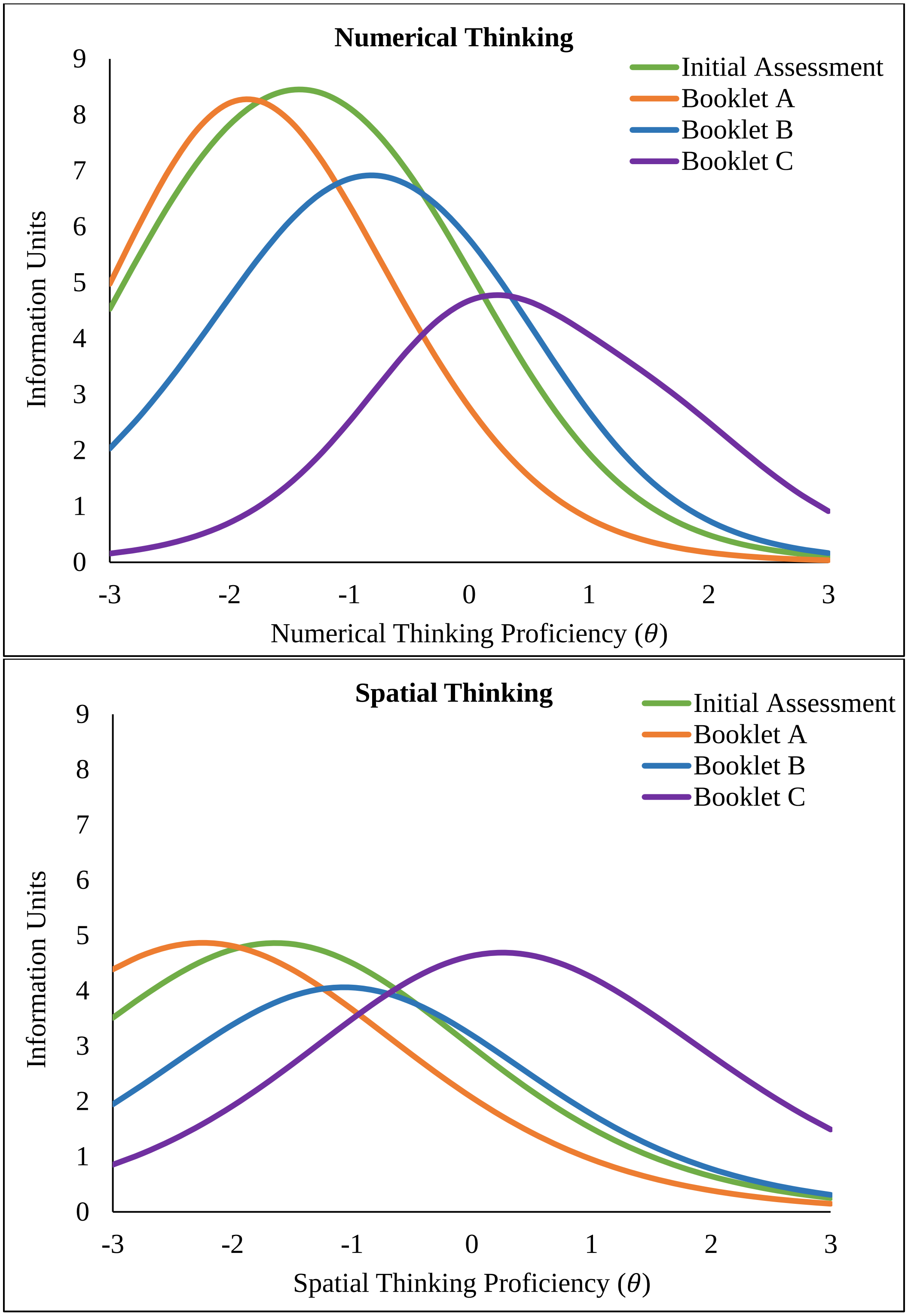

Figure 1. Information curves given latent proficiency for numerical thinking and spatial thinking assessment booklets.

A. Linear Trajectory View

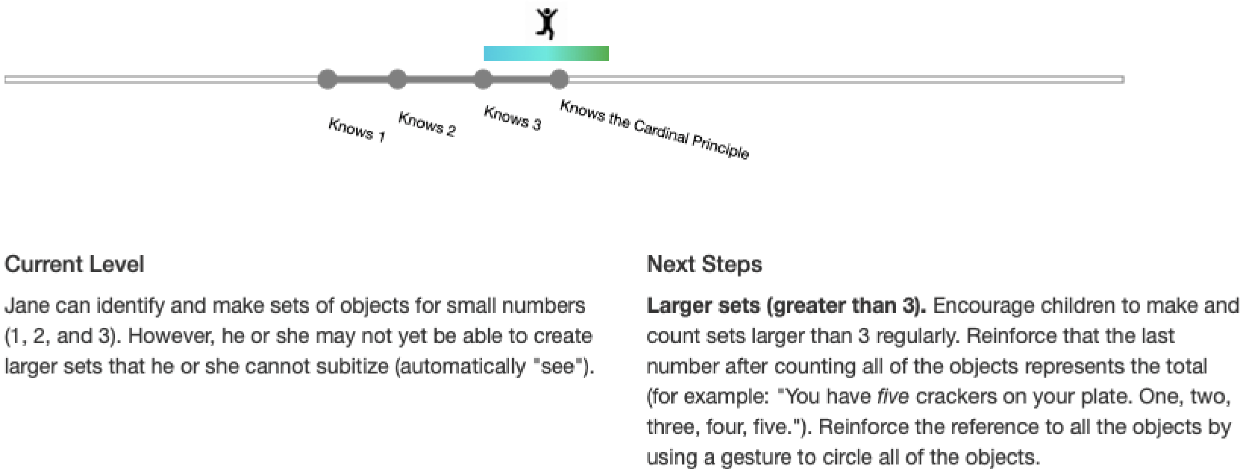

B. Growth Trajectory View

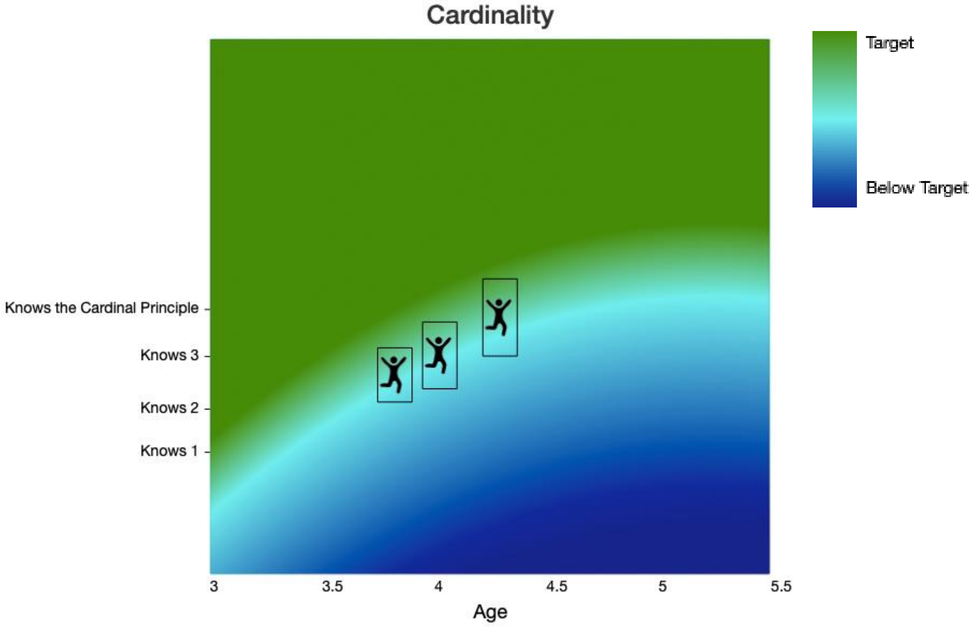

Figure 2. Example of feedback provided to teachers on an individual student's Cardinality proficiency
